# The Oligomeric States of the Purified Sigma-1 Receptor Are Stabilized by Ligands*

**DOI:** 10.1074/jbc.M113.537993

**Published:** 2014-05-20

**Authors:** Katarzyna A. Gromek, Fabian P. Suchy, Hannah R. Meddaugh, Russell L. Wrobel, Loren M. LaPointe, Uyen B. Chu, John G. Primm, Arnold E. Ruoho, Alessandro Senes, Brian G. Fox

**Affiliations:** From the ‡ Transmembrane Protein Center,; Departments of § Biochemistry and; ¶ Neuroscience, University of Wisconsin-Madison, Madison, Wisconsin 53706

**Keywords:** Ligand-binding Protein, Membrane Protein, Protein-Protein Interaction, σ Receptor, Signal Transduction, GXXXG Motif

## Abstract

Sigma-1 receptor (S1R) is a mammalian member of the ERG2 and sigma-1 receptor-like protein family (pfam04622). It has been implicated in drug addiction and many human neurological disorders, including Alzheimer and Parkinson diseases and amyotrophic lateral sclerosis. A broad range of synthetic small molecules, including cocaine, (+)-pentazocine, haloperidol, and small endogenous molecules such as *N,N*-dimethyltryptamine, sphingosine, and steroids, have been identified as regulators of S1R. However, the mechanism of activation of S1R remains obscure. Here, we provide evidence *in vitro* that S1R has ligand binding activity only in an oligomeric state. The oligomeric state is prone to decay into an apparent monomeric form when exposed to elevated temperature, with loss of ligand binding activity. This decay is suppressed in the presence of the known S1R ligands such as haloperidol, BD-1047, and sphingosine. S1R has a G*XXX*G motif in its second transmembrane region, and these motifs are often involved in oligomerization of membrane proteins. Disrupting mutations within the G*XXX*G motif shifted the fraction of the higher oligomeric states toward smaller states and resulted in a significant decrease in specific (+)-[^3^H]pentazocine binding. Results presented here support the proposal that S1R function may be regulated by its oligomeric state. Possible mechanisms of molecular regulation of interacting protein partners by S1R in the presence of small molecule ligands are discussed.

## Teaser

**Background:** Sigma-1 receptor (S1R) is an integral membrane ligand-binding receptor.

**Results:** Gel filtration chromatography revealed oligomeric states that are stabilized by ligand binding and destabilized by mutations in the G*XXX*G integral membrane dimerization domain.

**Conclusion:** Purified S1R binds small molecule ligands as an oligomer but not as a monomer.

**Significance:** The results provide new insight into the function of S1R with ligands and proteins partners.

## Introduction

The mammalian sigma-1 receptor (S1R)^6^ is a unique 223-amino acid membrane-bound protein (1–5). S1R is found in the mammalian central nervous system (CNS) and in most peripheral tissues, including the immune and endocrine systems. It is primarily localized in the endoplasmic reticulum (6–8) but also in some cellular plasma membranes (9), specialized cisternae-laden cholinergic synapses of the spinal cord ventral horn motoneuron C termini (10, 11), and in spinal cord dorsal root ganglia (12). The amino acid sequence of S1R is ∼95% identical between mammals, including the guinea pig, mouse, rat, and human. ERG2, a sterol isomerase found in yeast (13) and fungi (2), is an ortholog of the mammalian S1R with an approximately overall 30% sequence homology and 66% homology in the putative S1R ligand binding domain (1). The mammalian S1R does not possess sterol isomerase activity and has been clearly differentiated by sequence and size from the fungal sterol isomerase (1, 13).

S1R functions as a molecular chaperone and serves as a partner for a variety of client proteins. It stabilizes the inositol 1,4,5-trisphosphate type 3 receptor (14) in endoplasmic reticulum mitochondrial associated membranes and has been shown to interact and play an important regulatory role in many cell signaling systems, including the molecular chaperone GRP78/BIP (15), several types of G-protein-coupled receptors (15–18), and voltage-gated ion channels (9, 19–23). S1R suppresses the production of reactive oxygen species in various mouse tissues, including the retina, lung, and liver, and in cultured mammalian cells possibly by activating antioxidant response element genes (6, 24–26).

A broad range of synthetic small molecules with widely varied structures bind with high affinity to S1R, including the (+)-isomer of benzomorphan derivatives such as pentazocine and dextrallorphan, neuroleptics such as haloperidol, fluphenazine, and chlorpromazine, the compounds *o-*ditolylguanidine, PRE-084, BD-1047, and BD-1063, the beta-blocker propranolol, and the presynaptic dopamine D_2_ agonist (+)-3-PPP (27–29). Several endogenous small molecules such as *N,N*′-dimethyltryptamine (30), sphingosine (31), and steroids such as progesterone (32) and dehydroepiandrosterone (33) have also been identified as regulators of S1R.

Because of the broad contributions of S1R in maintaining cellular homeostasis, the receptor has been identified as a therapeutic target for the treatment of cancer (34) and neurodegenerative diseases, including amyotrophic lateral sclerosis (35), Alzheimer (36), and Parkinson diseases (37), and for retinal neurodegeneration (38). Several studies have also connected S1R to the possible treatment of drug addiction and toxicity related to derivatives of cocaine and amphetamine (8, 16, 39, 40).

The guinea pig S1R has been purified to homogeneity following expression in *Escherichia coli* as a fusion to the maltose-binding protein (MBP) (41). The ligand binding region of S1R was identified by the use of specific radioiodinated photoprobes (42–45) and by mutagenesis (46, 47) to be formed primarily by the juxtaposition of a short C-terminal hydrophobic region (residues 176–194) with a portion of TM2 (residues 91–109) and perhaps a portion of TM1. Based on hydrophobicity analyses and the use of S1R-GFP constructs (9) and S1R antibody probes (14), it has been concluded that the S1R contains two putative transmembrane (TM) helices (9) with both the N and C termini occurring on the cytoplasmic side of the cellular membrane (9, 14). S1R also has a G*XXX*G motif in TM2. This motif is often involved in helix-helix oligomerization of integral membrane proteins (48–50). High molecular weight forms (tetramer and pentamer) of the S1R were previously identified using radioiodinated photoaffinity labeling in rat liver microsomal membrane preparations (44), suggesting that S1R may oligomerize under physiological conditions.

Here, we report that highly purified S1R forms an oligomeric state, and we also show that the oligomeric state provides specific ligand binding, although the monomeric state does not. Stabilization of the functional oligomeric states occurs via the participation of the G*XXX*G oligomerization motif. These results are discussed in the context of possible mechanisms of molecular regulation of interacting protein partners by S1R in the presence of small molecule ligands.

## EXPERIMENTAL PROCEDURES

### 

#### 

##### Cloning

Plasmid DNA containing the guinea pig sigma-1 receptor gene was used as the template for all cloning work (41). All oligonucleotides were purchased from IDT (Coralville, IA). The MBP-4A-S1R plasmid used in this work was made using PIPE mutagenesis (51) as reported previously (52). PCR was carried out using Pfu-UltraII polymerase. When the PCR was completed, a DpnI digestion was performed to remove the template. The DpnI-digested PCR product was purified using a Qiagen PCR purification kit, and the eluted DNA was transformed into *E. coli* 10G (Lucigen, Middleton, WI).

To make expression plasmids for the second transmembrane helix (TM2) for TOXCAT analysis, two partially complementary long oligonucleotides corresponding to the S1R-TM2 domain were designed to include 5′ NheI and 3′ BamHI overhangs. These single-stranded oligonucleotides were allowed to anneal, and the resulting dsDNA was ligated into NheI- and BamHI-digested pccKan (53). Correct DNA constructs were verified by DNA sequencing of the entire MBP to ToxR fusion coding region. Mutations in TM2 were made using PIPE mutagenesis.

##### Protein Preparation

Expression and purification of MBP-4A-S1R containing a stabilizing 4-Ala linker between the MBP and S1R domains, a variant with a tobacco etch virus protease site present as the interdomain linker (MBP-TEV-S1R), or with mutations in TM2 were carried out as described previously (52). MBP-TEV-S1R was purified using amylose affinity chromatography (52) and subjected to proteolysis using TEV protease in a ratio of 1 mg of protease per 1 mg of fusion protein. TEV protease was prepared as reported previously (54). The TEV protease reaction was performed at room temperature for 96 h, and the final cleavage efficiency was greater than 95%. The sample was filtered through a 0.8-μm syringe filter and diluted with 20 mm Tris, pH 7.2, containing 0.031% Triton X-100 and 1 mm 2-mercaptoethanol to reduce the concentration of NaCl to 100 mm. A 5-ml Fast Flow HiTrap Q column (GE Healthcare) was prepared using 5 column volumes of 20 mm Tris, pH 7.2, containing 100 mm NaCl, 0.031% Triton X-100, and 1 mm 2-mercaptoethanol (loading buffer). The protein sample was loaded onto the Q column using the AKTA purifier sample pump at a flow rate of 1 ml/min and then washed with 5 column volumes of 20 mm Tris, pH 7.2, containing 100 mm NaCl, 0.031% Triton X-100, and 1 mm 2-mercaptoethanol (wash buffer). Elution was performed with a gradient of NaCl to a final concentration of 1 m over 20 column volumes. The collected fractions were analyzed for protein content by 4–20% gradient SDS-PAGE. Appropriate fractions were combined and concentrated as described before. Protein concentrations were determined using Bio-Rad in-gel densitometry. Samples from all purification steps were assayed for ligand binding activity.

##### Preparative Size Exclusion Chromatography

This chromatography was conducted on a Shimadzu Prominence HPLC equipped with a DGU-20A_5_ on-line degasser, LC-20AD solvent delivery module, SIL-20ACHT autosampler, CTO-20AC column oven, SPD-20A UV-vis detector, RF-10AXL spectrofluorometric detector, RID-10A differential refractometric detector, FRC-10A fraction collector module, CBM-20A system controller, and LabSolution LCsolution software version 1.24 SP1. The mobile phase, 10 mm HEPES, pH 7.2, containing 150 mm NaCl, 0.3 mm tris(2-carboxyethyl)phosphine, and 0.018% DDM (2× critical micelle concentration), was degassed for a minimum of 20 min under vacuum prior to use. The buffer was isocratically pumped at 1 ml/min through a Phenomenex 300 × 7.8-mm Yarra 3 μm SEC-3000 column with SecGuard column guard. Protein elution was monitored by UV absorption at 280 and 260 nm. The column temperature was 20.0 °C, and the detector flow cell temperature was 35.0 °C. Columns were calibrated daily using bovine thyroglobulin, IgA, ovalbumin, myoglobin, and uridine as standards (Phenomenex). High volume separation was achieved through repeated 100-μl injections while separating the protein into 41 125-μl fractions.

##### Analytical Size Exclusion Chromatography

Fractions from multiple injections of MBP-4A-S1R were subjected to additional rounds of analytical sizing chromatography to assess whether changes in the distribution of oligomeric states occurred during the repeat chromatography. Elution from the repeat chromatography was monitored using the intrinsic fluorescence of tryptophan with excitation at 280 nm and emission at 340 nm. During the course of the repeated analyses, retention times varied by less than 0.25 min during the 12-min chromatographic run (2%) and were corrected for preparation of figures to correspond to the same apparent molecular weight (determined from the daily calibration) by using the earliest chromatogram as the benchmark for retention times. Similarly, S1R obtained from TEV protease proteolysis of MBP-TEV-S1R was subjected to repeat sizing chromatography. In this case, the 41 proteolyzed fractions were stored for ∼1 month at 4 °C before repeat analysis.

##### Light Scattering Measurements

Separate fractions containing peaks 1 and 2 and an intermediate oligomer from Fig. 2*A* were subjected to size exclusion chromatography coupled to multiple angle laser light scattering, with UV absorbance and refractive index detection. Separation was performed on a Superdex S200 (GE Healthcare) in 150 mm NaCl, 5 mm HEPES, pH 7.2, and 0.018% DDM (2× critical micelle concentration). Light scattering and refractive index were measured with a DAWN HELEOS II and OPTILab rEX respectively (Wyatt Technology). Data analysis was performed using ASTRA 6.1 (Wyatt Technology).

##### Chemical Cross-linking

Chemical cross-linking with disuccinimidyl suberate (DSS) was performed on the detergent-solubilized and highly purified individual oligomeric states of MBP-4A-S1R. DSS was dissolved in DMSO, and the control samples (no cross-linker) contained equivalent amount of DMSO (2%). 5 μm of each protein state was incubated with either 30 or 50 m excess of DSS (150 and 250 μm, respectively) for 2 h at room temperature, in the presence or absence of 10 μm BD-1047. The reactions were stopped by addition of Tris-HCl, pH 8.0, to the final concentration of 30 mm. The samples were then subject to SDS-PAGE in 7.5% Tris-HCl Bio-Rad gel and calibrated with commercial molecular weight markers (Spectra Multicolor High Range Protein Ladder, Thermo Scientific). After staining with Coomassie Brilliant Blue R, the gels were imaged and analyzed using the GelAnalyzer 2010 free software to calculate the approximate molecular weight of visualized bands.

##### Oligomer Stability Tests

Haloperidol, *o-*ditolylguanidine, PRE-084, BD-1047, 4-PPBP, SKF-83959 (6-chloro-7,8-dihydroxy-3-methyl-1-(3-methylphenyl)-2,3,4,5-tetrahydro-1*H*-3-benzazepine), sphingosine, and sphingosine 1-phosphate were from Tocris Biosciences (Bristol, UK). Pentazocine was from Sigma. Stock solutions of PRE-084 (5 mm) and BD-1047 (10 mm) were prepared in deionized water. A pentazocine stock solution (10 mm) was prepared in 20 mm HCl in deionized water, and stock solutions of 4-PPBP (10 mm), haloperidol (10 mm), ortho-di-tolylguanidine (10 mm), and SKF-83959 (10 mm) were prepared in 100% (v/v) DMSO. Sphingosine and sphingosine 1-phosphate stock solutions (5 mm) were prepared in solution containing 1.8% (w/v) DDM. The sphingosine 1-phosphate stock solution was heated to ∼60 °C to aid in solubilization. Less than 0.5 mm free P_i_ was detected in this sample, indicating the phosphoryl group was not hydrolyzed during solubilization.

Purified peak 1 (see Fig. 2*B*) from preparative size exclusion chromatography of MBP-4A-S1R was diluted to 15 μg/ml (0.23 μm) and a final volume of 100–300 μl in 10 mm HEPES, pH 7.2, containing 0.3 mm tris(2-carboxyethyl)phosphine and 0.018% DDM and incubated for up to 18 h at 4, 25, and 37 °C. Analytical size exclusion chromatography was run before and after incubation with various ligands. A typical HPLC injection was 10 μl, and intrinsic tryptophan fluorescence was monitored. All experiments included a control sample incubated under the same conditions but with no added ligand. Ligand stock solutions were prepared as described above, and ligands were tested for stabilization at 0.45 and 10 μm.

##### Ligand Binding Assays

(+)-[^3^H]Pentazocine (specific activity 36 Ci/mmol) was from PerkinElmer Life Sciences. Binding assays were performed in 100 μl in a 48-well block format as described previously (41, 45) with minor modifications. Protein samples at 1 ng/μl were prepared in 20 mm Tris, pH 8.0, containing 0.1% Triton X-100. The final concentration of (+)-[^3^H]pentazocine in both total and nonspecific binding assays was 100 nm. Haloperidol (Tocris) was used as the masking agent in the nonspecific binding reaction at final concentration of 10 μm. The incubation with ligands was performed for 90 min at 32 °C, followed by filtration on a glass fiber filter (Whatman GF/B) performed in a Brandel cell harvester. The glass filter was then washed with 50 mm Tris, pH 8.0, and individual filters were transferred into vials containing 3 ml of scintillation mixture (Ultima Gold, PerkinElmer Life Sciences). The level of radioactivity was measured the following day using a Packard scintillation counter. The raw count data were normalized to nanomoles of protein present in the assay and plotted as the percentage of specific binding activity of the original control sample MBP-TEV-S1R.

The stoichiometry of ligand binding was determined using 300 nm of purified peak 1 (see Fig. 2*B*) supplemented with a range of concentrations of BD-1047 from 0 to 3000 nm in a total volume of 150 μl. The titration was assembled in a 96-well plate and incubated for 16 h at 37 °C. Aliquots (10 μl) from each well were examined with analytical size exclusion chromatography, and eluted protein was detected using tryptophan fluorescence. The values for 0 and 100% oligomeric stabilization were normalized using the wells containing either no BD-1047 or the maximal amount, respectively. The binding data were analyzed using *K_D_* = [*n*P][L]/[PL], where *n* is the number of protein molecules that bind one molecule of ligand, and P, L, and PL are the equilibrium concentrations of free receptor, free ligand, and the ligand-bound receptor, respectively. The expression for *K_D_* was rewritten as *K_D_* = (*n*P_i_ − *x*)(L_i_ − *x*)/(*x*), where *x* corresponds to the amount of [PL] formed and also the depletion in concentrations of free receptor and free ligand; P_i_ indicates initial protein; and L_i_ indicates initial ligand. Theoretical values for *x* at each step in the ligand binding titration were determined by solving this latter expression. Best fit values for *n* and *K_D_* were determined using the NonlinearModelFit routine of Mathematica version 8.0.4.0 (Wolfram Research).

##### TOXCAT

A gene encoding the TM2 domain of S1R was cloned into the NheI-BamHI restriction sites of the pccKAN vector resulting in the following sequence, NRAS*XXX*GILIN. *E. coli* MM39 cells transformed with pccKan-derived TOXCAT plasmids were inoculated into 3 ml of Luria Bertani medium containing 100 μg/ml ampicillin and grown overnight at 37 °C with shaking. To check for proper membrane insertion of the TOXCAT constructs, overnight cultures of transformed MM39 cell were plated onto M9 minimal medium agar plates containing 0.4% maltose as the only carbon source and grown at 37 °C for 48 h (53). Aliquots (3 μl) of the overnight cultures were inoculated into 3 ml of Luria Bertani medium and grown to *A*_600_ of 0.6 at 37 °C with shaking. An aliquot (1 ml) of the culture medium was centrifuged for 10 min at 17,000 × *g,* and the cell pellet was resuspended in 500 ml of 25 mm Tris-HCl, pH 8.0, containing 2 mm EDTA. The resuspended cells were sonicated at medium power for 10 s, and a 50-μl aliquot was removed from each sample and mixed with 4× NuPAGE SDS loading buffer, boiled for 10 min, and saved for Western blotting. Lysates were clarified by centrifugation at 17,000 × *g* for 10 min. The supernatant was kept on ice and used in chloramphenicol acetyltransferase (CAT) assays.

##### Chloramphenicol Acetyltransferase Assays

One ml of 0.1 m Tris-HCl, pH 7.8, containing 0.1 mm acetyl-CoA and 0.4 mg/ml 5,5′-dithiobis-(2-nitrobenzoic acid) was mixed with 40 μl of supernatant from the cell lysis, and the absorbance at 412 nm was measured for 2 min to establish basal activity (55). After this, 40 μl of 2.5 mm chloramphenicol dissolved in 10% ethanol was added, and absorbance at 412 nm was measured for an additional 2 min to determine CAT activity. CAT activity was normalized using *A*_420_ measurements of cell aliquots. The relative CAT activities were reported as percentages of the activity given by the strong transmembrane dimer control, glycophorin A.

##### Quantification of TOXCAT Expression

Boiled cell lysates (10 μl) were loaded onto a NuPAGE 4–12% BisTris SDS-polyacrylamide gel (Invitrogen) and then transferred to polyvinylidene fluoride membranes (VWR) for 1 h at 100 mV. Blots were blocked using 5% bovine serum albumin (US Biologicals) in 50 mm Tris, pH 8.0, containing 150 mm NaCl and 0.5% Tween buffer (TBST) for 2 h at 4 °C. Biotinylated anti-maltose-binding protein antibody (Vector Laboratories) was diluted 1:1500 in 1% bovine serum albumin in TBST and incubated overnight at 4 °C. Blots were washed with TBST for 1 h with three buffer exchanges at room temperature before incubation with secondary antibody in 1% bovine serum albumin in TBST at 1:1500 dilution, peroxidase-conjugated streptavidin (Jackson ImmunoResearch) for 2 h at room temperature. Blots were again washed for 1 h using three exchanges of TBST. A 1:1 mixture of buffers from the Pierce ECL Western blotting substrate kit was added to the blot, and chemiluminescence was measured using an ImageQuant LAS 4000 (GE Healthcare).

## RESULTS

**FIGURE 1.**
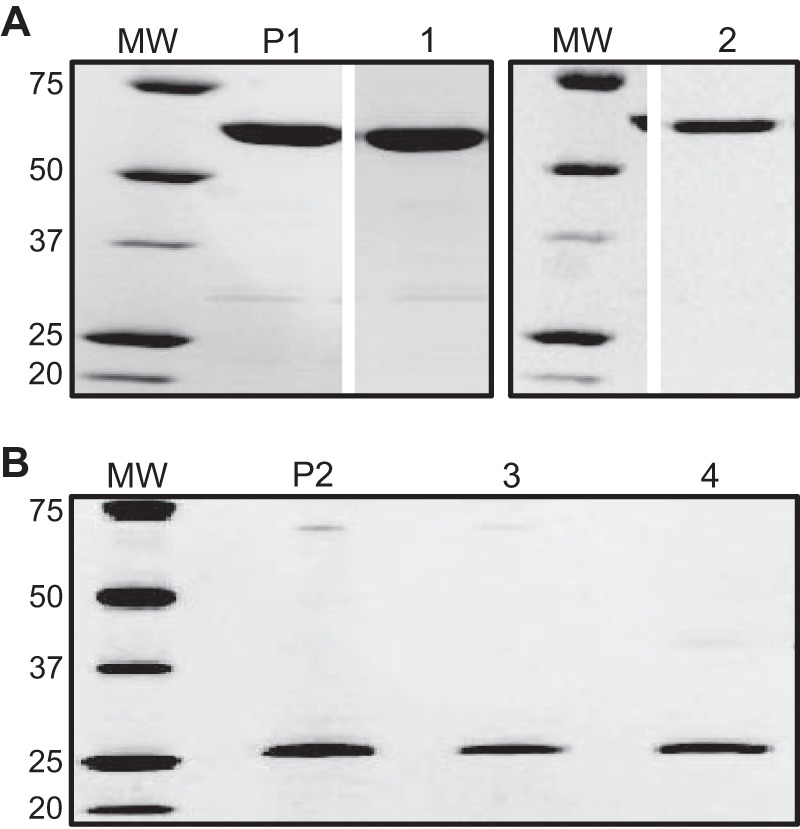
**SDS-PAGE of MBP-4A-S1R (*A*) and S1R (*B*).** The starting MBP-4A-S1R and S1R preparations prior to SEC separation are labeled *P1* and *P2*, respectively. *Peaks 1–4* from Fig. 2*A* are labeled respectively. Molecular mass markers (*MW*) are labeled in kDa.

For these studies, MBP-4A-S1R (Fig. 1*A*, *lane P1*) was prepared using an expression method that gives higher yield of purified protein from *E. coli* (52). In addition, S1R without an MBP tag was prepared by treatment of a MBP-TEV-S1R fusion with TEV protease (Fig. 1*B*, *lane P2*). Denaturing SDS-PAGE showed that these protein preparations consisted of a single polypeptide with purity greater than 95%. With these preparations, we investigated the relationship between the oligomerization state of S1R and its ligand binding activity. The results show that an oligomeric form of the receptor is required for specific ligand binding.

### 

#### 

##### Evidence for Oligomerization

**FIGURE 2.**
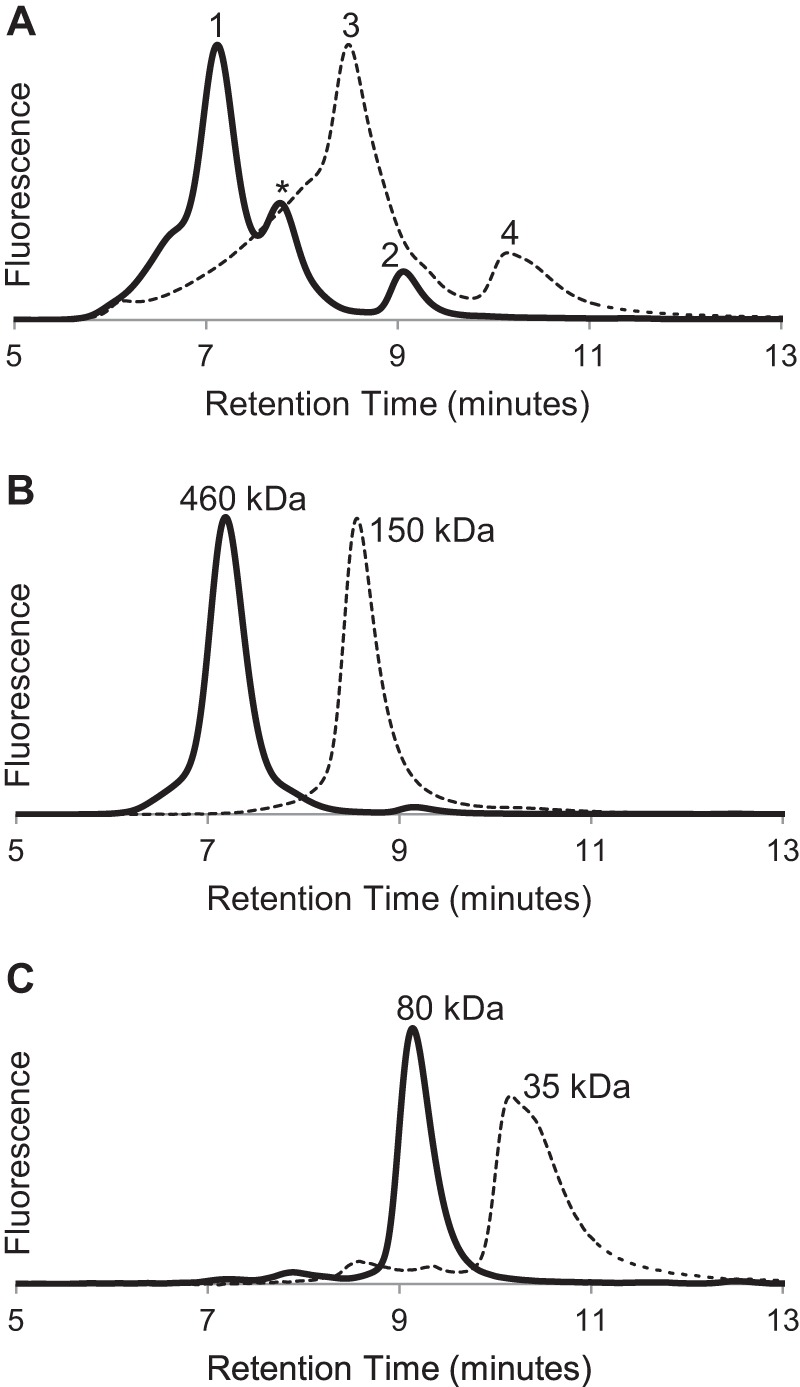
**Evidence from size exclusion chromatography for oligomeric states of S1R.** *A,* elution profiles for MBP-4A-S1R (*solid line*) and S1R (*dashed line*). Peaks corresponding to different oligomerization states are marked as *1–4. B,* repeat chromatography of peaks 1 (*solid line*) and 3 (*dashed line*) from *A. C,* repeat chromatography of peaks 2 (*solid line*) and 4 (*dashed line*) from *A*.

Analytical size exclusion chromatography of purified MBP-4A-S1R showed two major peaks, labeled *1* and *2* (*solid line,* Fig. 2*A*). After treatment with TEV protease, purified S1R also showed two major peaks, labeled *3* and *4* (*dotted line,* Fig. 2*A*). Thus, both receptor preparations show evidence for formation of a predominant oligomeric state (peaks 1 and 3) along with a corresponding monomer (peaks 2 and 4). Similar behavior was observed from MBP-4A-S1R prepared in buffer containing *n*-octyl-β-d-glucopyranoside or MBP-4A-S1R prepared in buffer containing Triton X-100. A variable amount of an intermediate oligomer state was also observed in the MBP-4A-S1R samples (retention time ∼8 min, marked with * in Fig. 2*A*). Fig. 2, *B* and *C,* shows that both the oligomer and monomer peaks were stable (*i.e.* eluted with the same retention time) when subjected to a repeat round of chromatography. Thus, the major peaks shown in Fig. 2*A* could be obtained in a highly pure form. Fig. 2*B* shows that peaks 1 and 3, corresponding to the potential oligomeric states, had apparent molecular masses of 460 and 150 kDa, respectively, for the protein-detergent micelle, although Fig. 2*C* shows that peaks 2 and 4, corresponding to monomeric states, had apparent molecular masses of 80 and 35 kDa, respectively. The oligomeric assemblies were only dependent on the presence of S1R, as MBP alone did not form an oligomeric state in the conditions used here. Moreover, after removal of the MBP by treatment with TEV protease, S1R remained in the oligomeric state and could be further purified by both adsorption and size exclusion chromatographies (Fig. 2*B*, *peak 3*).

**FIGURE 3.**
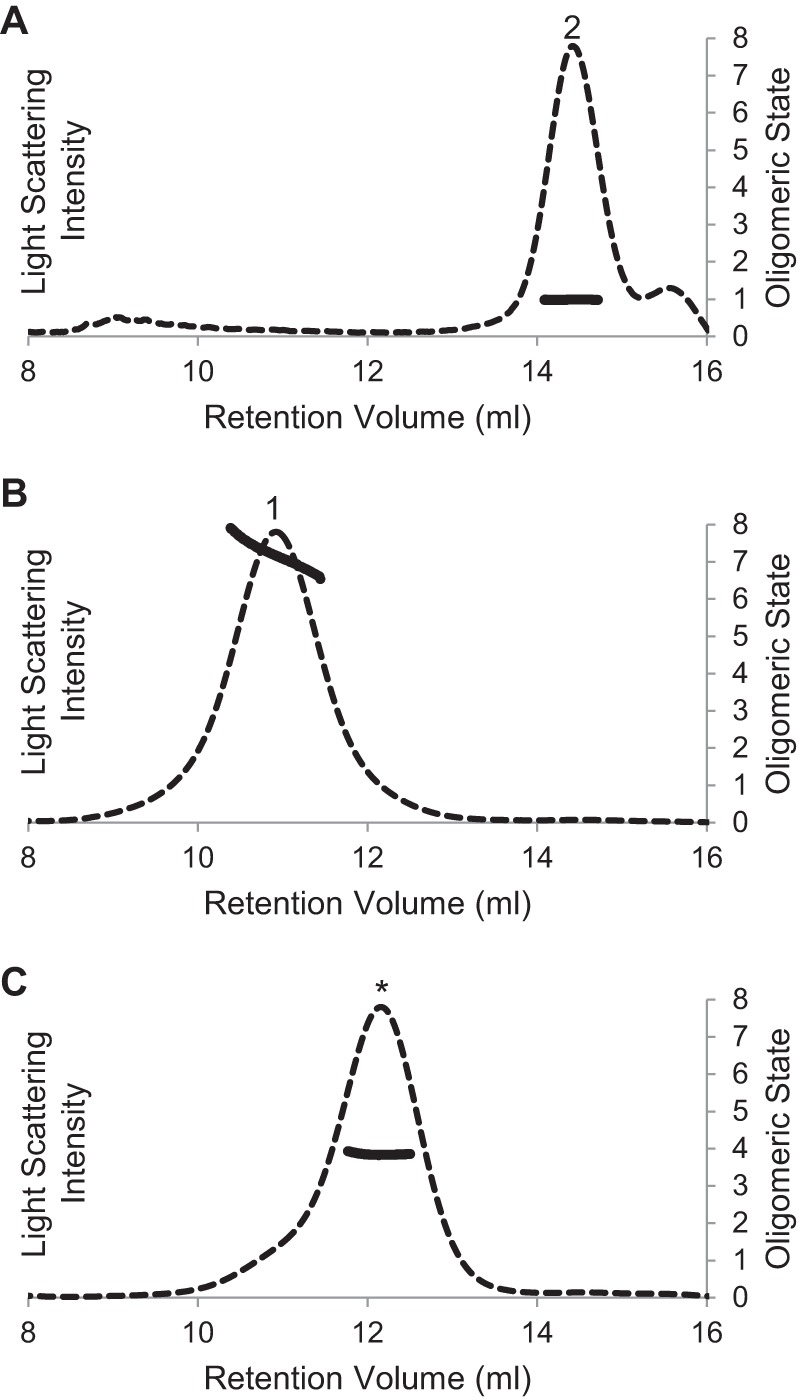
**Oligomeric molecular weight of MBP-4A-S1R determined by light scattering.** Elution profiles detected by 652-nm laser light scattering are shown by *dashed lines*. Oligomeric stoichiometry across each peak is marked with a *solid line*. Peaks have the same labeling as in Fig. 1 and 2. *A,* apparent monomer is confirmed to be a monomer. *B,* largest molecular weight oligomer is polydisperse, with stoichiometry ranging from a hexamer to octamer. *C,* intermediate oligomer is a tetramer.

The estimation of oligomer stoichiometry using analytical SEC alone is complicated because of uncertainty in how the protein will be accommodated into a protein-detergent micelle and those effects on the hydrodynamic radius. Although static light scattering measurements are often used to assess oligomeric stoichiometry, the presence of detergent micelles creates high background noise and must be accounted for in the mass of the protein-detergent complex. Thus, the fusion protein was sent to the Yale Keck Biophysics Lab, which is specially equipped for these measurements, with combined SEC and light scattering instrumentation. Three peaks were analyzed. The smallest protein molecules detected (Fig. 3*A*) had a mass within 2% of that predicted for a monomer, whereas the largest molecular weight protein (Fig. 3*B*) was polydisperse, with molecular weights corresponding to oligomerization states of 6–8. Light scattering also showed that the intermediate oligomer marked with * in Fig. 2*A* was monodisperse with a molecular weight corresponding to a tetramer (Fig. 3*C*).

**FIGURE 4.**
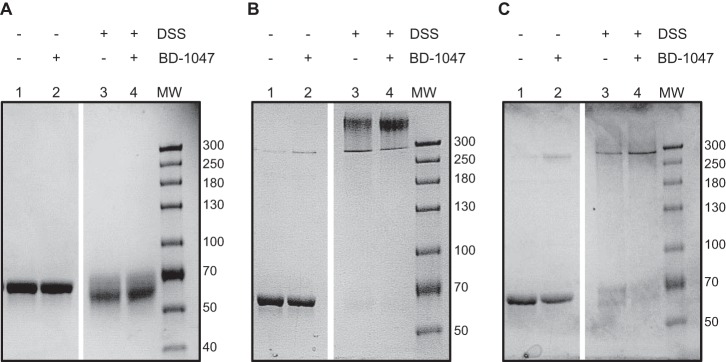
**Analysis of oligomeric states of MBP-4A-S1R by chemical cross-linking agent DSS.** DSS-free controls are shown by *lanes 1* and *2*, containing 0 and 10 μm high affinity ligand BD-1047, respectively. *Lanes 3* and *4* mirror the control with the addition of DSS. *A,* addition of cross-linking agent to the monomer (Fig. 2*A, peak 2*). The bands show slight smearing with no shift in size, signifying only intramolecular cross-linking. *B,* addition of cross-linking agent to the oligomer (Fig. 2*A*, *peak 1*). The bands show a mass greater than 300 kDa, and oligomeric stoichiometry cannot be accurately determined. However, an oligomeric state greater than tetramer is clearly visible. *C,* addition of cross-linking agent to the intermediate oligomer (Fig. 2*A, peak* *). The bands show an approximate 4-fold increase in size to a mass between 250 and 300 kDa, suggesting a tetrameric state.

Analysis by SDS-PAGE after cross-linking gave further insight into the light scattering results. Fig. 4*A* shows the monomer was unchanged in SDS-PAGE either without cross-linker (*lanes 1* and *2*) or with cross-linker (*lanes 3* and *4*), suggesting no intermolecular interactions capable of being captured by the cross-linking reagent. In contrast, Fig. 4*B* shows that the polydisperse oligomer cross-linked to a size greater than 300 kDa. Furthermore, Fig. 4*C* shows that the intermediate oligomer, assigned to be a tetramer by light scattering measurements, was cross-linked to a molecule with molecular weight again consistent with a tetramer.

##### Ligand Binding

**FIGURE 5.**
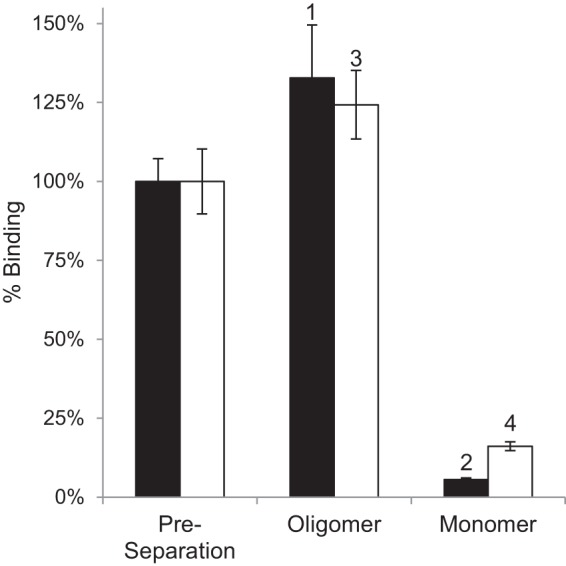
**Comparison of specific pentazocine binding activity of S1R oligomers before and after repeat chromatography of peaks from Fig. 2, *B* and *C*.** The *black bars* are for assays of MBP-4A-S1R; *white bars* are for assays of S1R. Binding assays were performed in triplicate, and the *error bars* represent 1σ deviation.

Pentazocine is a well studied ligand for S1R (56, 57). Fig. 5 shows that only the oligomeric states of MBP-4A-S1R and S1R exhibited specific pentazocine binding activity. For example, peak 1 from Fig. 2 (oligomer of the MBP fusion) bound pentazocine with ∼20× higher specific activity than peak 2 (monomer of the MBP fusion). Likewise, the specific binding activity for peak 2 from Fig. 3 (S1R oligomer) was ∼15× higher than for peak 4 (S1R monomer). Because some S1R ligands were delivered in the DMSO carrier, a sample containing a final concentration of 2% DMSO but no other ligand was tested independently and shown to have no effect on the oligomeric state. With these assignments of the active form of the receptor, peak deconvolution of the original samples indicated that active MBP-4A-S1R (*solid line,* Fig. 2, *A* and *B*) was ∼75% of the original protein sample, although active S1R (*dotted line,* Fig. 2, *A* and *B*) represented ∼50%.

Ligand binding was also tested using the fluorescent ligand BODIPY^TM^-sphingosine in analytical SEC experiments. Consistent with the specific pentazocine binding results (Fig. 5), the oligomeric forms of the protein migrated with haloperidol-masked BODIPY fluorescence, but the monomer did not (data not shown). The fluorescent ligand was a gift from Mary L. Kraft Ph.D. (University of Illinois, Urbana-Champaign).

##### Stability of Oligomers

**FIGURE 6.**
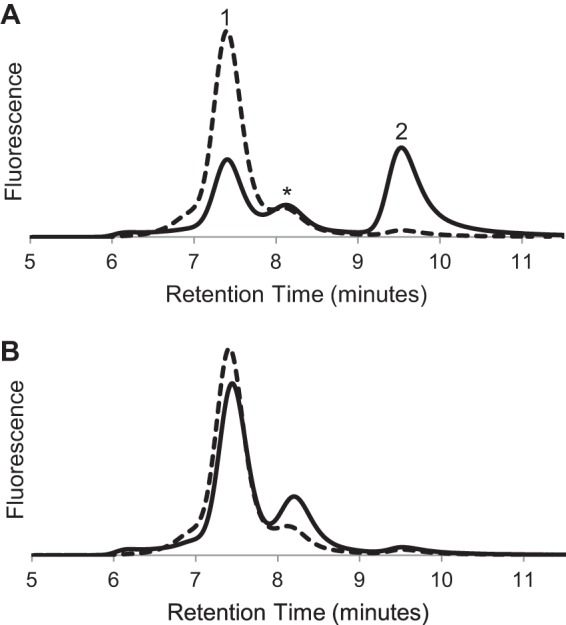
**Stabilization of oligomeric S1R by ligand binding.** *A,* size exclusion chromatogram of MBP-4A-S1R before (*dashed line*) and after incubation for 1 day at 37 °C (*solid line*) without added ligand. Peaks have the same labeling as in Fig. 1 and 2. *B,* chromatogram of MBP-4A-S1R before (*dashed line*) and after (*solid line*) incubation for 1 day at 37 °C in the presence of the tight binding ligand BD-1047 (0.45 μm).

The oligomeric forms of S1R were observed to decay upon incubation in buffer. Thus, after ∼18 h at 37 °C, the amount of protein in peak 1 decreased by ∼40%, and the amount in peak 2 increased by a corresponding amount (Fig. 6*A*). Similar instability of the S1R oligomers was also observed at 25 and 4 °C in the absence of ligand but to a lesser extent. In all chromatograms, the total integrated area remained constant within 5%, supporting the conclusion that interconversion between oligomer and monomer states was occurring. Disulfide bonds are apparently not involved in oligomer formation because inclusion of 100 mm 2-mercaptoethanol in the buffer used for S1R purification and repeat analytical size exclusion chromatography did not change the elution profile.

**FIGURE 7.**
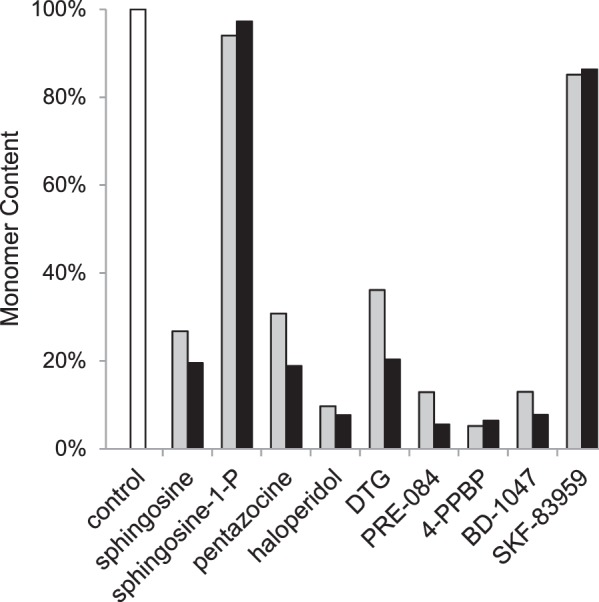
**Comparison of the ability of various S1R ligands to prevent conversion to the inactive monomer state.** The amount of MBP-4A-S1R converted to the monomer (*peak 2* from Fig. 2) in the absence of ligands served as the control (*white bar*). *Gray* and *black bars* indicate ligand doses of 0.45 and 10 μm, respectively, whereas the concentration of MBP-4A-S1R was always 0.23 μm. Tight-binding ligands 4-PPBP, BD-1047 and others stabilized the oligomeric states, whereas sphingosine 1-phosphate allowed conversion to the monomeric state.

Fig. 6*B* shows that in the presence of 0.45 μm BD-1047, a known tight-binding ligand of S1R, the relative proportions of peaks 1 and 2 were essentially unchanged after the ∼18-h incubation; similar behavior was observed at each of the three temperatures tested. Several other known S1R ligands, including the natural membrane constituent sphingosine, also gave stabilizing interactions. Fig. 7 shows the percentage increase in the monomer form after incubation with these ligands relative to a control containing no ligand, where a larger increase in monomer corresponds to less effective stabilization of the active oligomer. Overall, the tightest binding synthetic ligands (*e.g.* BD-1047 and 4-PPBP) gave the largest stabilizing effect (*i.e.* least conversion to the monomer). Interestingly, the natural membrane lipid sphingosine gave a stabilizing interaction that was close to that of many of the synthetic ligands. In contrast, another natural membrane lipid, sphingosine 1-phosphate, did not stabilize the receptor even when present at greater than 20× higher concentration than BD-1047 (10 μm *versus* 0.45 μm).

**FIGURE 8.**
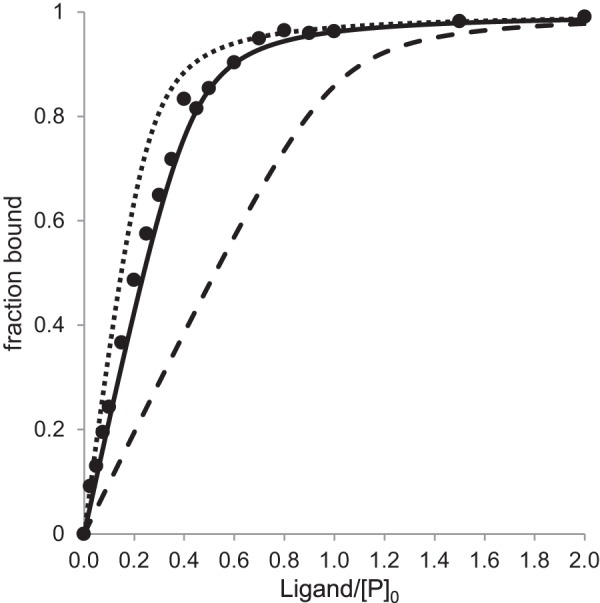
**Ligand binding stoichiometry determined by titration of BD-1047 into a 300 nm sample of peak 1 (see Fig. 2*B*).** Experimental measurements (*solid circles*) were made in the concentration range from 0 to 3000 nm, with results shown up to 600 nm ligand. Binding curves were calculated as described under “Experimental Procedures” with best fit values of *K_D_* = 7 nm and *n* = 0.43 (*solid line*), fixed *K_D_* = 7 nm and *n* = 0.25 (*dotted line*), or fixed *K_D_* = 7 nm and *n* = 1 (*dashed line*).

Fig. 8 shows an analysis of stoichiometry of binding for BD-1047, a tight-binding ligand, as assessed by stabilization of the peak 1 oligomer. In the experiment with receptor-detergent micelles carried out here, the best fit *K_D_* (*r*^2^ = 0.998, *solid black line*) was ∼7 nm, which is comparable the value reported elsewhere (58). The best unconstrained fit stoichiometry of ligand bound per receptor, *n*, was determined to be 0.43, *i.e.* corresponding to ∼1 mol of ligand per 2 mol of receptor. Fits with *n* held constant at 1 were not compatible with the binding data (Fig. 8, *dashed line*), as acceptable fits could not be obtained at any *K_D_* value. Moreover, when *n* was held constant at 0.25, approximate fits using the best-fit *K_D_* values gave systematic overestimation of the fraction bound at a low ligand/protein ratio (Fig. 8, *dotted line*). An analysis assuming *n* was 0.33, *i.e.* ligand binds to a trimer, also gave a similar overestimation; increasing the *K_D_* value gave a better fit at low ligand/protein ratios but gave under-estimation of complex formation at high ligand/protein ratios. An identical experiment was conducted on the intermediate oligomer (Fig. 2*A*, *peak labeled* *) yielding the same results.

##### Role of GXXXG Motif in Oligomerization

**FIGURE 9.**
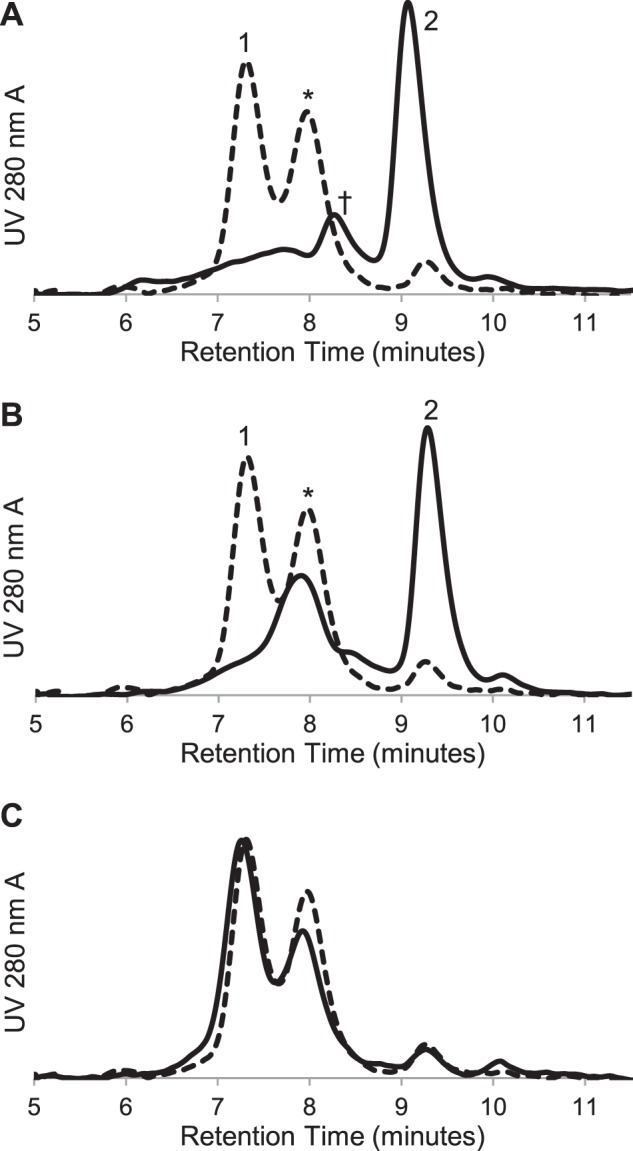
**Size exclusion chromatography of MBP-4A-S1R with mutations in the G*XXX*G motif.** The control chromatogram of MBP-4A-S1R lacking mutations is shown as a *dotted line. A,* G91I MBP-4A-S1R with defined peaks as in Fig. 2. A significant shift toward the monomeric state is seen, as is a new ∼180 kDa peak labeled with †. *B,* G91L MBP-4A-S1R showing conversion to intermediate oligomeric (*) and monomer (*2nd peak*) states. *C,* H97A MBP-4A-S1R showed little change in the oligomerization states relative to the nonmutated receptor.

The G*XXX*G motif is often involved in helix-helix association in integral membrane proteins (49, 59). In S1R, TM2 contains this motif in the primary sequence Gly-87–Gly-88–Trp-89–Met-90–Gly-91. Mutations of the Gly residues in this motif and an adjacent His residue in MBP-4A-S1R were prepared to test their roles in oligomerization. All mutations within the motif (substitutions of either Ile or Leu at Gly-87, Gly-88, and Gly-91) resulted in lower expression and significantly decreased yield for the purified fusion protein. For example, MBP-4A-S1R gave a purified yield of ∼3.5 mg per liter of culture medium, whereas G91I MBP-4A-S1R (∼0.4 mg/liter) and G91L MBP-4A-S1R (∼0.6 mg/liter) were the best yields for the proteins with mutations in the G*XXX*G motif. With all of the purified receptor variants, all mutations in the G*XXX*G motif eliminated the largest oligomer (*e.g. peak 1* in Fig. 2) from size exclusion chromatographs. Fig. 9 shows representative behavior for mutation of Gly-91 to either Ile (Fig. 9*A*) or Leu (Fig. 9*B*). Both mutations strongly shifted the distribution of receptor states to the monomer form (Fig. 9, *A* and *B, solid lines*). With the G91I mutation (Fig. 9*A*), a smaller oligomeric state (retention time ∼8.2 min, marked with †) with an apparent molecular mass of ∼180 kDa was observed. All Gly-88 and Gly-89 mutants had similar chromatograms. This peak plausibly represents a dimeric state of the S1R. Fig. 9*C* shows that the mutation H97A, which is not in the G*XXX*G motif, yielded a chromatograph almost identical to MBP-4A-S1R, suggesting this residue does not play an important role in oligomerization.

**FIGURE 10.**
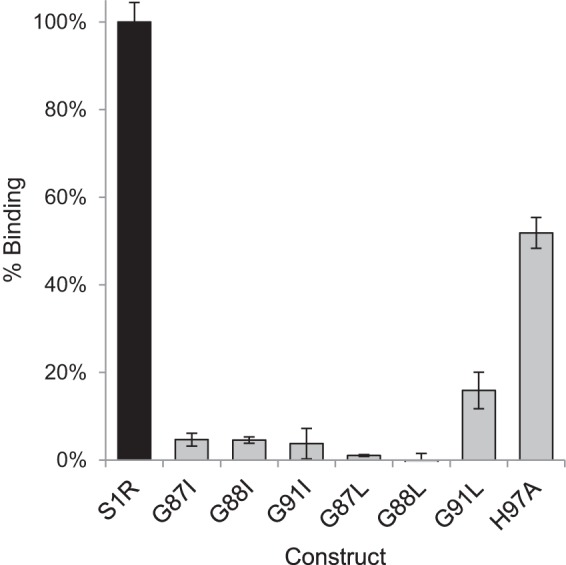
**[^3^H]Pentazocine-specific binding for MBP-4A-S1R and the variants with mutations in the G*XXX*G motif.** Binding activity is shown relative to MBP-4A-S1R (*black bar*); *n* = 3; *error bars* represent 1σ deviation.

Fig. 10 shows that mutations within the G*XXX*G motif had a profound effect on specific ligand binding. Among the set of all G*XXX*G mutations, G91L MBP-4A-S1R exhibited ∼20% of specific pentazocine binding activity of the nonmutated receptor, although all other mutations in the G*XXX*G motif yielded purified receptor variants that had less than 5% specific binding. Corresponding to the modest level of specific binding activity observed, G91L MBP-4A-S1R uniquely stabilized a significant fraction of the purified protein as the intermediate oligomer (Fig. 9*B*), likely tetramer (Fig. 3*C*). These results further implicate the role of an oligomer in ligand binding. Although H97A MBP-4A-S1R had a distribution of large, intermediate, and monomer states that was nearly identical with the nonmutated fusion protein (Fig. 9*C*), it exhibited only ∼50% ligand binding activity (Fig. 10). This result suggests a role for His-97 in ligand binding independent of oligomerization.

##### Oligomerization of TM2

**FIGURE 11.**
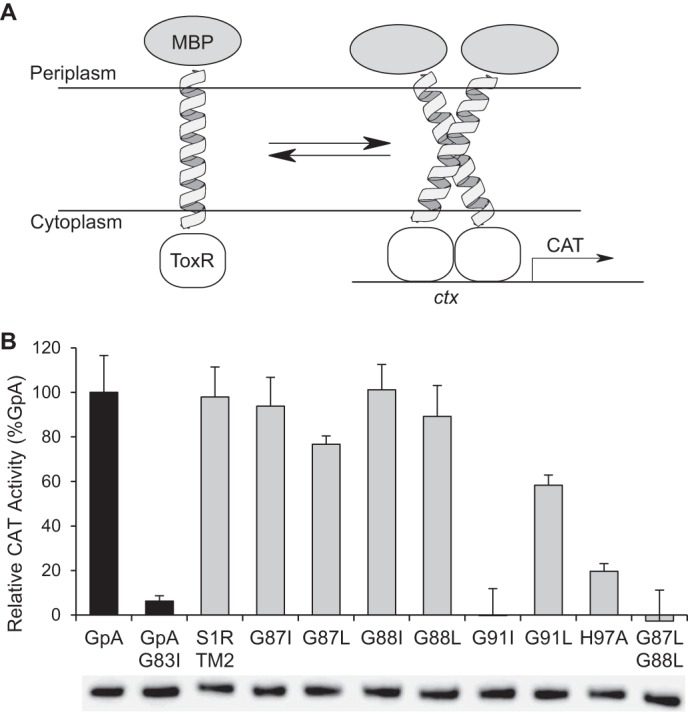
**TOXCAT measurements for mutations of the TM2 domain of S1R.** *A,* schematic of the TOXCAT experiment, where periplasmic secretion of MBP is used to place a TM domain into the cytoplasmic membrane, whereas ToxR resides in the cytoplasm. Dimerization of the TM promotes dimerization of ToxR, which then binds to the ctx promoter acting as a transcriptional activator, in this case for CAT. *B,* TOXCAT response is reported as a percentage relative to the strong transmembrane oligomerization control, glycophorin A (*GpA*). Immunoblot results obtained from anti-MBP-HRP are shown below the *histogram bars* and indicate equivalent expression of all TM2 domain variants.

To further assess the propensity of TM2 for self-association, TOXCAT reporter assays were performed by inserting the sequence of TM2 (WVFVNAGGWMGAMCLLHASL) between MBP and the ToxR receptor (Fig. 11*A*). With this construct, oligomerization of the TM2 region can be assessed by catalytic assay of the ToxR-mediated expression of CAT, as ToxR only functions as a transcriptional enhancer when it is present as an oligomer. Fig. 11*B* shows results from the TOXCAT experiment. All variants were expressed to a comparable level as assessed by Western blotting, and nonmutated TM2 from S1R gave rise to a strong positive CAT assay response, corroborating the propensity of the TM2 sequence to self-associate. Interestingly, among the single mutations of the G*XXX*G motif, only G91I eliminated the positive response in the CAT assay, indicating this mutation strongly destabilized oligomerization of the TM2. Surprising, the G91L mutation gave a positive assay response, albeit attenuated to only 60% of that observed from the nonmutated TM2. Individually, mutations at either Gly-87 or Gly-88 did not affect the assay response. Although this result is apparently contradictory to the results of Fig. 10 obtained with the full-length receptor, the adjacent positions of Gly-87 and Gly-88 in the primary sequence of the TM2 peptide presumably allowed alternative modes for association of the TM2 peptide that could not be achieved with the TM2 present in the MBP-4A-S1R fusion. The double mutation G87L/G88L eliminated the TOXCAT assay response, perhaps corresponding to more effective disruption of alternative TM2 interactions leading to oligomerization.

## DISCUSSION

In this work, we provide biochemical evidence for the importance of an oligomeric state in the ligand binding function of guinea pig S1R and the essential role of the G*XXX*G motif from TM2 in this oligomerization. Because amino acid sequences and pharmacological profiles are highly conserved among mammalian S1R, these results are likely broadly relevant to many studies of this protein family (60).

Mutational analysis of putative TM2 in both the full-length receptor and as a transmembrane domain in the TOXCAT studies have identified key residues involved in oligomerization (Gly-87, Gly-88, and Gly-91) and in ligand binding independent of oligomerization (His-97). This work adds to the list of other residues in TM2 (Ser-99, Tyr-103, and Leu-105–Leu-106) that are important for ligand binding (61), which this work suggests is intimately related to the ability to form one or more oligomeric states. Although the G*XXX*G motif within TM2 has an important role in oligomer formation, other studies have implicated additional residues of the S1R receptor sequence in dimerization/oligomerization (17), which could explain the appearance of the 180-kDa peak (Fig. 9*A, peak* †). The S1R ligand-binding site in the intact receptor, as identified by photoaffinity labeling, has been localized to a region that juxtaposes a steroid binding domain-like motif (SBDLI) in TM2 (which encompasses the oligomerization sequences identified in this work) and a C-terminal SBDLII hydrophobic sequence (42–44). Although the C terminus alone does not bind S1R ligands, some of the chaperone functions of S1R have been localized to this region (62). It has been proposed that S1R agonists alter the structure of the receptor in such a fashion that the C-terminal chaperone region becomes accessible to its client proteins. NMR-derived structures of the C terminus have been recently reported (63). Perhaps the oligomeric states of the S1R receptor dictate the availability of the C terminus for these interactions (64).

Several previous experiments support the conclusion that S1R functions as an oligomer in ligand binding. For example, different molecular weights have been observed by gel filtration chromatography for S1R purified from natural sources (65). Moreover, high molecular weight bands of S1R protected from photoaffinity labeling by (+)-pentazocine were detected by denaturing gel electrophoresis of rat liver microsomes (44, 64). Furthermore, gel filtration of ligand-bound S1R purified from human leukemia cells showed that ligand binding activity was associated with a protein of ∼100 kDa (2). Many other membrane receptors adopt an oligomeric state (66–68), and recombinant expression often leads to a distribution of these states (69). For example, when human serotonin receptor 3A was overexpressed in *E. coli*, the protein was detected as a mixture of oligomers, and the biologically active pentamer represented only 7% of the total protein (70). The percentage of active S1R protein determined in the study reported here was between 50 and 75%.

S1R interacts with a large number of synthetic and natural ligands, and it has also been documented to interact with a large number of different proteins within the cell (5, 30). We found that interactions with the tightest binding synthetic ligands strongly stabilize the oligomeric state (Figs. 6 and 7). The stabilizing effects of ligands on many other membrane proteins, including G-protein-coupled receptors, are recognized (71, 72). Our studies with purified S1R indicate a minimal binding stoichiometry of one ligand per two polypeptide chains (Fig. 8), although reconciliation of gel filtration, light scattering, and denaturing gel electrophoresis results obtained with purified S1R suggests the octamer, hexamer, and tetramer are the predominant ligand-binding forms. A stoichiometry of one ligand bound/dimer is further supported by the work of Chu *et al.* (64), who showed that a C12 alkyl containing photoprobe selectively and quantitatively derivatized His-145 at a proposed S1R dimer interface. Furthermore, dimerization of S1R, as assessed by SDS-PAGE, occurred via intermolecular disulfide bond formation when an M170C mutant form of the receptor was expressed.

**FIGURE 12.**
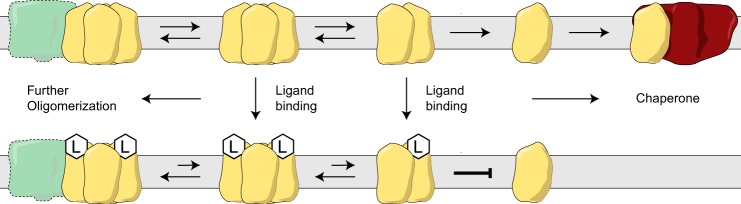
**Model representing the proposed interconversions of S1R between the monomer form and ligand-stabilized oligomeric forms.** Protein partners of the monomer form would include voltage-gated Na^+^ channel, acid-sensing channels, and dopamine D1 receptor (16, 23, 73).

The oligomer can decay to an intermediate tetramer and monomer in the absence of ligands (Fig. 6), although mutations of the G*XXX*G motif change the distribution of these species. Preliminary efforts to reassemble the monomer into functional oligomers were not successful. However, it is intriguing to consider that protein-protein interactions may be involved in the reassembly of the functional receptor. In this regard, monomeric S1R has been reported to interact with ion channels such as Nav 1.5 voltage-gated Na^+^ channel, acid-sensing channels, and the dopamine D_1_ receptor (16, 23, 73). Interestingly, these interactions were disrupted in the presence of ligands such as haloperidol and/or (+)-pentazocine, so one may speculate that a ligand-gated S1R oligomer/monomer equilibrium defines the availability of monomer S1R for interaction with client ion channels or G-protein-coupled receptors. An additional unusual feature of binding of the agonist, (+)-pentazocine, to S1R is the time (>90 min at 30 °C) needed to reach equilibrium (41, 74). It is possible that formation of stable interactions between oligomeric states of S1R *in situ* regulate the rate of (+)-pentazocine binding to S1R (perhaps also affected by interactions with accessory protein partners). Fig. 12 provides a schematic of these possibilities.

## Footnotes

6 The abbreviations used are:

S1R: sigma-1 receptor
BD-1047: *N*-[2-(3,4-dichlorophenyl)ethyl]-*N*-methyl-2-(dimethylamino)ethylamine dihydrobromide
MBP: maltose binding protein
4-PPBP: 4-phenyl-1-(4-phenylbutyl)piperidine maleate
PRE-084: 2-(4-morpholinethyl)-1-phenylcyclohexanecarboxylate hydrochloride
TM2: second transmembrane region
ToxR: *Vibrio cholerae* toxin transcriptional regulator
BisTris: 2-[bis(2-hydroxyethyl)amino]-2-(hydroxymethyl)propane-1,3-diol
DSS: disuccinimidyl suberate.
